# AI-assisted 3D preoperative planning in primary total hip arthroplasty for secondary hip osteoarthritis: etiology-specific perioperative burden and early recovery in DDH versus post-septic sequelae

**DOI:** 10.3389/fmed.2026.1805046

**Published:** 2026-03-30

**Authors:** Zhenbao Lu, Tihui Wang, Xu Wang, Yuhua Feng, Xiaohong Fan, Qiujin Xia, Jiliang Chen, Hongkuan Lin, Chengshou Lin, Qingshan Xu, Qijin Wang

**Affiliations:** 1 Department of Orthopaedics, Affiliated Mindong Hospital, Fujian Medical University, Ningde, Fujian, China; 2 School of New Energy and Intelligent Manufacturing, Ningde Vocational and Technical College, Ningde, Fujian, China

**Keywords:** 3D planning, artificial intelligence, developmental dysplasia of the hip, post-septic hip osteoarthritis, total hip arthroplasty

## Abstract

**Background:**

Computed tomography (CT)-based, artificial intelligence (AI)-assisted three-dimensional (3D) preoperative planning is increasingly used to standardize component sizing and positioning in total hip arthroplasty (THA). In complex secondary hip osteoarthritis (HOA), perioperative burden and early recovery are particularly relevant to older or medically complex patients, yet it remains unclear whether etiology still shapes these outcomes under a unified planning pathway and single implant system.

**Methods:**

This single-center retrospective cohort analyzed 95 consecutive cementless primary THAs for developmental dysplasia of the hip-associated HOA (DDH-HOA, *n* = 52) or post-septic HOA (*n* = 43), all performed by one senior surgeon using the same CT-based AI-assisted 3D planning pathway and one cementless implant system. The primary endpoint was the Harris Hip Score (HHS) at 24 months; secondary outcomes included operative time, intraoperative blood loss (IBL), femoral osteotomy, planning-to-implant concordance, radiographic targets, patient-reported outcome measures (PROMs), and complications.

**Results:**

Planning performance was comparable (cup match 59.62% vs. 65.12%; stem match 61.54% vs. 67.44%), with similar Lewinnek/Callanan safe-zone attainment and a median leg-length discrepancy (LLD) of 1 mm in both groups. Intraoperative blood loss was lower in DDH-HOA than in post-septic HOA (212.69 ± 62.31 vs. 243.72 ± 58.64 mL, *P* = 0.015), while operative time was comparable (72.31 ± 14.11 vs. 77.91 ± 14.56 min, *P* = 0.061) and femoral osteotomy rates did not differ. At 1 month, DDH-HOA showed better pain and function [visual analogue scale (VAS) *P* = 0.035; HHS *P* < 0.001; Western Ontario and McMaster Universities Osteoarthritis Index (WOMAC) *P* = 0.004], whereas by 24 months HHS was comparable (*P* = 0.062). Two sciatic nerve injuries occurred in post-septic HOA; no reoperation or revision was recorded through 24 months.

**Conclusion:**

Under a unified CT-based AI-assisted 3D planning pathway and single cementless system, sizing concordance and radiographic target attainment were similarly high across etiologies. By 24 months, PROMs were broadly comparable between groups. Post-septic sequelae were associated with greater intraoperative blood loss and less favorable pain and function at 1 month, supporting etiology-informed counseling and targeted perioperative risk mitigation.

## 1 Introduction

Hip osteoarthritis (HOA) is a leading cause of chronic hip pain and functional limitation, and its global disability burden continues to rise (1). As arthroplasty demand increases in aging healthcare systems, perioperative burden and early recovery after total hip arthroplasty (THA) have become increasingly consequential, as blood loss and delayed mobilization can translate into greater care needs and healthcare resource utilization. Among secondary causes, developmental dysplasia of the hip (DDH) is associated with earlier onset and faster progression of HOA (2), and patients therefore often undergo THA at a relatively young age (3). In contrast, long-term sequelae of septic arthritis may leave persistent structural destruction and deformity, predisposing to secondary degenerative changes and ultimately necessitating THA in a subset of patients to relieve pain and restore function (4, 5). In complex reconstructions such as adult high hip dislocation, DDH-associated HOA and post-septic hip osteoarthritis (post-septic HOA) represent two common etiological categories, both characterized by abnormal anatomy and compromised soft tissues that can complicate preoperative planning and intraoperative execution (6, 7).

Although both entities can achieve substantial pain relief and functional improvement after THA, the perioperative challenge profile differs by etiology. In DDH-associated HOA, difficulty is driven mainly by osseous dysmorphology and the need to restore the hip center within the true acetabulum. This is most evident in Crowe III–IV high dislocations, where subtrochanteric femoral shortening osteotomy is often required to facilitate reduction while mitigating neural traction risk and aiding correction of leg-length discrepancy (LLD) and overall limb alignment (6, 8). Reported functional gains after THA in DDH can approach those observed in primary HOA, although revision risk remains a concern in subgroups with severe deformity and high dislocation (3). In contrast, post-septic HOA is frequently accompanied by bone loss and dense periarticular scarring with contracture (9). Even when infection has remained adequately quiescent and primary THA is considered appropriate (10), surgical exposure and reduction may still be demanding, and intraoperative blood loss (IBL) and reconstructive burden can be more pronounced (4, 5). Accordingly, even among patients with a similar degree of dislocation, etiological differences may influence operative time, IBL, and the need for intraoperative femoral osteotomy, thereby shaping early recovery trajectories and perioperative resource utilization, particularly in older or medically complex patients (6, 7).

Computed tomography (CT)-based, artificial intelligence (AI)-assisted three-dimensional (3D) preoperative planning has emerged as a standardized and reproducible clinical decision-support tool for surgical planning in THA. By integrating 3D reconstruction with virtual trialing, this pathway enables preoperative simulation of component sizing and positioning and may improve the predictability and consistency of key reconstructive parameters (11–13). Systematic reviews indicate that AI-based or CT-3D planning improves implant size prediction compared with conventional two-dimensional, radiograph-based templating; however, evidence for perioperative metrics and patient-reported outcome measures (PROMs) remains mixed, likely reflecting case complexity, platform variability, and study heterogeneity (14). Existing studies have largely stratified outcomes by Crowe classification or deformity severity, whereas etiology-focused comparisons conducted within a unified CT-based, AI-assisted 3D planning pathway and implant system remain scarce (6, 15, 16). Consequently, it remains unclear whether etiology still influences perioperative burden and early recovery when THA is delivered through a standardized AI-assisted pathway.

Accordingly, this retrospective cohort study compared perioperative burden and postoperative outcomes between DDH-associated hip osteoarthritis (DDH-HOA) and post-septic hip osteoarthritis (post-septic HOA) within a unified CT-based, AI-assisted 3D planning pathway using a single cementless implant system. Outcomes included perioperative burden, planning-to-implant agreement, radiographic accuracy, and PROMs at 1 and 24 months, with the Harris Hip Score (HHS) at 24 months designated as the primary endpoint. It was hypothesized that early recovery would be faster in DDH-HOA, whereas post-septic HOA would be associated with greater perioperative burden and slower early improvement, with differences narrowing by two years, thereby informing etiology-sensitive counseling and perioperative resource planning.

## 2 Materials and methods

### 2.1 Study design and ethical considerations

This single-center retrospective cohort study compared etiology-specific perioperative burden and postoperative recovery after primary THA for DDH-HOA versus post-septic HOA. All cases were managed using the same CT-based, AI-assisted 3D preoperative planning pathway and a single cementless implant system. The study was conducted in accordance with the Declaration of Helsinki and reported in line with the Strengthening the Reporting of Observational Studies in Epidemiology (STROBE) statement. Ethical approval was obtained from the institutional ethics committee (Approval No. H20250801503). Written informed consent permitting the use of anonymized data for future research was obtained at the time of surgery.

### 2.2 Patient selection

Consecutive patients who underwent primary THA for end-stage symptomatic HOA between January 2019 and November 2023 were retrospectively screened. Etiology (DDH-HOA or post-septic HOA) was determined based on clinical history and imaging findings. Only cases with complete perioperative records and completion of the pre-specified 24-month assessment were included.

Inclusion criteria were: (1) primary THA for end-stage symptomatic HOA; (2) DDH-HOA or post-septic HOA supported by history and imaging; (3) availability of a preoperative CT scan adequate for AI-assisted 3D planning; and (4) availability of key outcomes at baseline and at 1 and 24 months postoperatively.

Exclusion criteria were: (1) active infection or suspected ongoing infection at the time of THA, or infection-driven staged procedures; (2) prior ipsilateral hip arthroplasty or major ipsilateral hip surgery substantially altering native anatomy; (3) alternative etiologies (e.g., osteonecrosis, inflammatory arthritis, fracture sequelae, tumor-related reconstruction); (4) CT datasets inadequate for 3D planning; and (5) missing key data or follow-up <24 months. For the post-septic HOA cohort, absence of active infection was defined clinically as no draining sinus, no recent antibiotic therapy for hip infection, and no clinical or laboratory evidence suggestive of ongoing infection.

### 2.3 Preoperative planning

All patients underwent CT-based, AI-assisted 3D preoperative planning using AIJOINT (Changmugu Medical Technology Co., Ltd., Beijing, China; version 1.0), which remained unchanged throughout the inclusion period. The software automatically generated pelvic and femoral segmentations from CT datasets using a deep convolutional neural network–based segmentation framework, as reported for hip CT segmentation in THA planning (17). The operating surgeon reviewed the segmentation and planning outputs for quality control; although manual refinement is available, the exported outputs were accepted as generated in this cohort (modification rate, 0%). The planning report recorded acetabular cup size, target inclination and anteversion, planned hip center, leg-length and offset targets, and femoral stem size and alignment. The final plan was confirmed by the senior surgeon, and intraoperative adjustments were permitted when required by exposure, bone quality, bone defects, or soft-tissue tension.

### 2.4 Surgical procedure

All procedures were performed by the same senior arthroplasty surgeon using a standardized posterolateral approach. After femoral neck osteotomy, acetabular preparation and cup implantation were performed aiming to reproduce the planned hip center and target orientation. Femoral preparation and stem implantation were then completed to restore planned offset and leg length. Cementless components were used in all cases, and hip stability was assessed intraoperatively.

Etiology-specific measures were applied when indicated. In the DDH-HOA cohort, true acetabular reconstruction and femoral realignment were prioritized, and subtrochanteric femoral shortening osteotomy was performed when necessary to facilitate reduction while avoiding excessive limb lengthening and mitigating neural traction risk. In the post-septic HOA cohort, additional soft-tissue release and debridement were performed as needed due to scarring and associated bone defects, and reconstruction was escalated when bony support or stability was deemed insufficient. Perioperative management followed a uniform institutional protocol in both groups, including antibiotic prophylaxis, blood conservation measures, standardized rehabilitation, and venous thromboembolism prophylaxis.

All procedures used a single cementless implant system (Pinnacle acetabular component and Summit femoral stem, DePuy Synthes, Warsaw, IN, United States).

### 2.5 Radiographic assessment

Preoperative dislocation severity was graded using the Crowe classification on standard anteroposterior (AP) pelvic radiographs. Postoperative cup inclination and anteversion were measured on standardized AP pelvic radiographs using Feitu imaging software (Zhejiang Feitu Imaging Technology Co., Ltd., China; version 2.19.6). Measurements were performed by two independent observers blinded to etiology. Cup orientation was categorized as within the Lewinnek and Callanan safe zones according to published thresholds (18, 19). LLD was assessed on calibrated AP pelvic radiographs using the inter-teardrop line as the pelvic reference and the lesser trochanter as the femoral landmark.

### 2.6 Data collection and outcome measures

Data were extracted from planning reports, operative notes, anesthesia records, and follow-up documentation. The primary endpoint was HHS at 24 months (20). Key secondary outcomes included perioperative burden, planning-to-implant agreement, radiographic accuracy, PROMs at 1 and 24 months, complications, and any reoperation or revision. Perioperative burden was defined by operative time, IBL, and the requirement for intraoperative femoral osteotomy, and these metrics were considered proxies for perioperative resource utilization. Planning accuracy was assessed using planning-to-implant size agreement for the acetabular cup and femoral stem, reported as both exact match and ±1 size agreement. Here, ±1 size agreement was defined as an implanted size identical to the planned size or differing by one size. PROMs were collected preoperatively and at pre-specified time points (1 and 24 months) using WOMAC (21), HHS, and a visual analogue scale (VAS) for pain (22). Completion of the 24-month assessment was required for inclusion.

### 2.7 Statistical analyses

Analyses were performed using IBM SPSS Statistics (version 26.0; IBM Corp., Armonk, NY, United States). Normality was assessed using the Shapiro–Wilk test. Normally distributed continuous variables are presented as mean ± standard deviation and were compared using the independent-samples *t*-test, with Welch’s *t*-test used when variances were unequal. Non-normally distributed data are presented as median (interquartile range) and were compared using the Mann–Whitney U test. Categorical variables are presented as counts (percentages) and were compared using the Chi-square test or Fisher’s exact test, as appropriate. PROMs were compared between groups at each time point without formal group-by-time interaction testing. Given the retrospective design, analyses were primarily unadjusted, with baseline characteristics reported to contextualize potential confounding. All tests were two-sided, and *P* < 0.05 was considered statistically significant.

## 3 Results

### 3.1 Baseline characteristics

After excluding five patients (two with incomplete records, two lost to follow-up, and one who did not consent to data use), 95 patients were analyzed (DDH-HOA, *n* = 52; post-septic HOA, *n* = 43). Baseline demographic and preoperative characteristics were comparable between groups, including age, sex, body mass index (BMI), American Society of Anesthesiologists (ASA) class, operative side, Dorr type, and Crowe type (all *P >* 0.05). Preoperative clinical status was also similar, including LLD, HHS, WOMAC, and VAS pain score (all *P >* 0.05) (Table 1).

**TABLE 1:** Baseline demographic and preoperative clinical characteristics.

Parameters	DDH-HOA group (*n* = 52)	Post-septic HOA group (*n* = 43)	*P*-value
Sex			
Female	32	25	0.736^b^
Male	20	18	
Age (years)	62.60 ± 11.14	61.47 ± 10.09	0.609^a^
BMI (kg/m^2^)	23.75 ± 2.93	24.12 ± 3.10	0.553^a^
ASA			
I–II	41	34	0.979^b^
III–IV	11	9	
Operative side			
Left	19	21	0.227^b^
Right	33	22	
>Dorr type			
A	35	29	0.989^b^
B	17	14	
Crowe type			
I–II	41	33	0.806^b^
III–IV	11	10	
VAS pain score	5.8 ± 1.5	6.2 ± 1.1	0.114^d^
HHS	51.33 ± 12.29	47.58 ± 9.36	0.104^a^
WOMAC	52.5 (48.0, 58.0)	55.0 (49.5, 62.0)	0.300^c^
LLD (mm)	9.0 (6.0, 15.5)	11.0 (8.0, 17.5)	0.147^c^
24-month assessment completed, *n* (%)	52 (100)	43 (100)	–

*P*-values: *t*-test (^a^) for Age, BMI and HHS; Welch’s t test (^d^) for VAS; Mann–Whitney U test (^c^) for WOMAC and LLD; Chi-square test (^b^) for categorical variables. ASA, American Society of Anesthesiologists; BMI, body mass index; DDH, developmental dysplasia of the hip; HOA, hip osteoarthritis; HHS, Harris Hip Score; LLD, leg-length discrepancy; VAS, visual analogue scale; WOMAC, Western Ontario and McMaster Universities Osteoarthritis Index.

### 3.2 Perioperative comparison

Operative time did not differ significantly between groups (DDH-HOA vs. post-septic HOA: 72.31 ± 14.11 vs. 77.91 ± 14.56 min; mean difference 5.60 min; *P* = 0.061). Intraoperative blood loss was higher in the post-septic HOA group (243.72 ± 58.64 vs. 212.69 ± 62.31 mL; mean difference 31.03 mL; *P* = 0.015). The requirement for intraoperative femoral osteotomy was comparable (11/52 vs. 13/43; *P* = 0.318) (Table 2).

**TABLE 2:** Perioperative surgical parameters in the DDH-HOA and post-septic HOA groups.

Parameters	DDH-HOA group (*n* = 52)	Post-septic HOA group (*n* = 43)	*P*-value
Operative time (min)	72.31 ± 14.11	77.91 ± 14.56	0.061^a^
Acetabular component exact match rate	59.62%	65.12%	0.583^b^
Acetabular component ± 1 size agreement	82.70%	86.05%	0.655^b^
Femoral stem exact match rate	61.54%	67.44%	0.550^b^
Femoral stem ± 1 size agreement	92.31%	95.35%	0.303^b^
Intraoperative blood loss, IBL (mL)	212.69 ± 62.31	243.72 ± 58.64	0.015^a^
Cup within Lewinnek safe zone	92.31%	93.02%	0.598^b^
Cup within Callanan safe zone	86.54%	88.37%	0.789^b^
LLD (mm)	1.0 (1.0, 2.0)	1.0 (1.0, 2.0)	0.242^c^
Intraoperative femoral osteotomy, *n*	11	13	0.318^b^

*P*-values: *t*-test (^a^) for Operative time and IBL; Mann–Whitney U test (^c^) for LLD; Chi-square test (^b^) for size agreement rates, safe-zone attainment, and femoral osteotomy. DDH, developmental dysplasia of the hip; HOA, hip osteoarthritis; IBL, intraoperative blood loss; LLD, leg-length discrepancy.

Planning-to-implant concordance and radiographic target attainment were similar across etiologies. Exact match rates were comparable for both acetabular cup size (59.62% vs. 65.12%; *P* = 0.583) and femoral stem size (61.54% vs. 67.44%; *P* = 0.550). When allowing a one-size deviation, ±1 size agreement remained high and comparable between groups for both components (Table 2). Cup orientation fell within the Lewinnek safe zone in 92.31% vs. 93.02% (*P* = 0.598) and within the Callanan safe zone in 86.54% vs. 88.37% (*P* = 0.789). Postoperative LLD was similar (median 1.0 mm in both groups; *P* = 0.242) (Table 2).

### 3.3 Postoperative outcomes

The primary endpoint, HHS at 24 months, was comparable between groups (DDH-HOA: 87.0 [85.0–90.0] vs. post-septic HOA: 85.0 [84.0–89.0], *P* = 0.062) (Table 3). At 1 month, DDH-HOA showed lower pain and better early function, with lower VAS (2.2 [1.6–4.3] vs. 3.0 [2.3–3.8], *P* = 0.035), higher HHS (75.25 ± 11.22 vs. 67.51 ± 10.20, *P* < 0.001), and lower WOMAC (23.29 ± 8.86 vs. 29.35 ± 10.99, *P* = 0.004). By 24 months, VAS and WOMAC were similar between groups (*P* = 0.443 and *P* = 0.329, respectively) (Table 3).

**TABLE 3:** Patient-reported outcomes and hip function at 1 and 24 months.

Parameters	DDH-HOA group (*n* = 52)	Post-septic HOA group (*n* = 43)	*P*-value
VAS at 1 month	2.2 (1.6, 4.3)	3.0 (2.3, 3.8)	0.035^c^
HHS at 1 month	75.25 ± 11.22	67.51 ± 10.20	<0.001^a^
WOMAC at 1 month	23.29 ± 8.86	29.35 ± 10.99	0.004^a^
VAS at 24 months	1.44 ± 0.48	1.52 ± 0.45	0.443^a^
HHS at 24 months	87.0 (85.0, 90.0)	85.0 (84.0, 89.0)	0.062^c^
WOMAC at 24 months	16.60 ± 3.73	17.40 ± 4.20	0.329^a^

*P*-values: *t*-test (^a^) for HHS at 1 month, WOMAC at 1 month, VAS at 24 months, and WOMAC at 24 months; Mann–Whitney U test (^c^) for VAS at 1 month and HHS at 24 months. DDH, developmental dysplasia of the hip; HOA, hip osteoarthritis; HHS, Harris Hip Score; VAS, visual analogue scale; WOMAC, Western Ontario and McMaster Universities Osteoarthritis Index.

### 3.4 Complications and reoperations

No deep infection, aseptic loosening, periprosthetic fracture, reoperation, or revision occurred in either group during the 24-month follow-up. Two patients in the post-septic HOA group experienced postoperative sciatic nerve injury, with deficits persisting at 24 months. Superficial wound exudation occurred in one patient in each group and resolved with local wound care.

### 3.5 Representative cases

To illustrate the CT-based, AI-assisted 3D planning pathway and the correspondence between planned and implanted components, one representative case from each cohort is presented.

#### 3.5.1 DDH-HOA case

**FIGURE 1:**
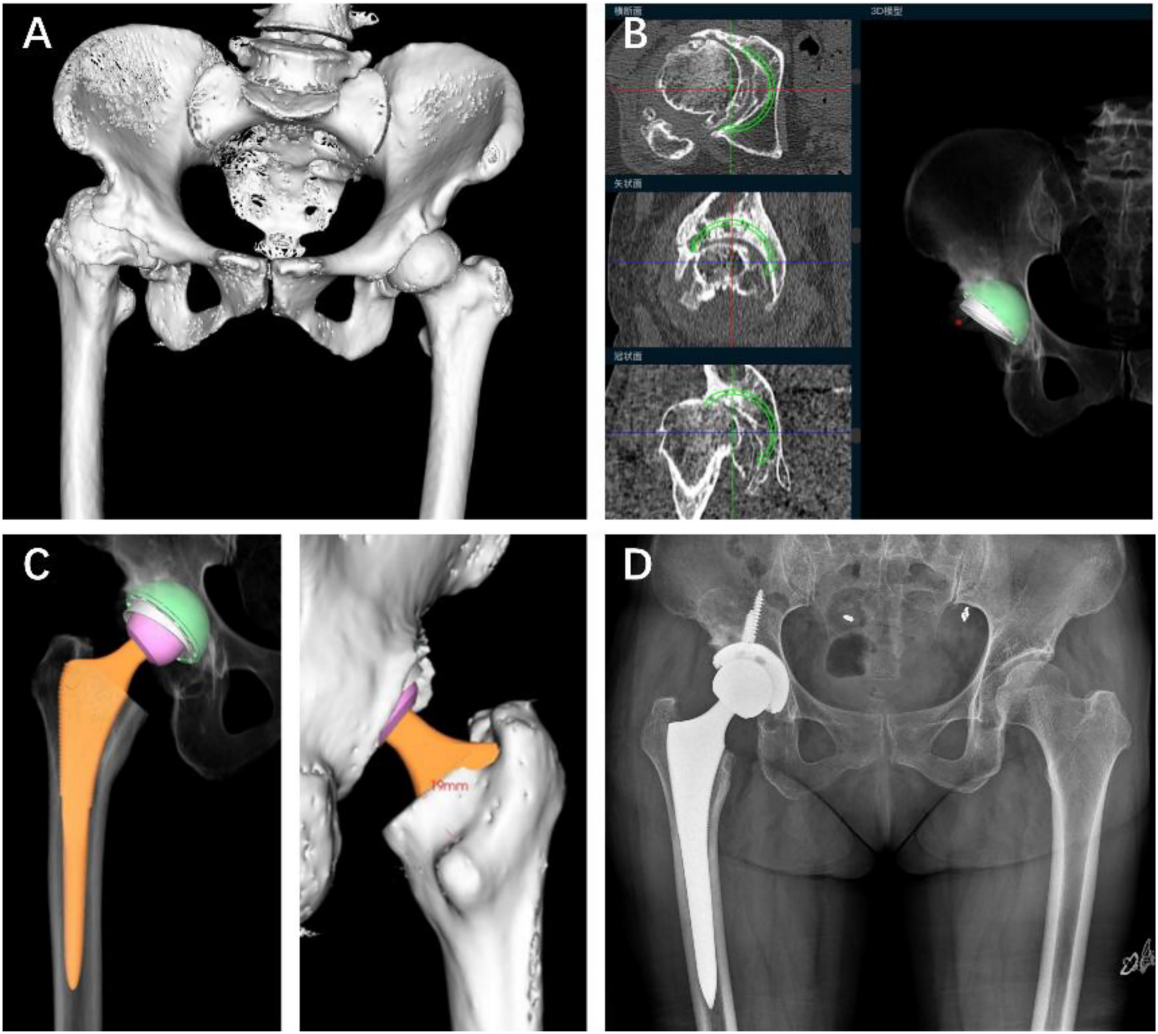
AI-assisted 3D preoperative planning in a Crowe II DDH-HOA case. **(A)** 3D CT reconstruction. **(B)** Planned acetabular cup (Pinnacle, size 50). **(C)** Planned femoral stem (Summit, size 6). **(D)** Postoperative radiograph showing the final position of the planned implants.

A 69-year-old woman underwent primary THA for DDH-HOA (Crowe type II). CT-based, AI-assisted 3D planning was performed to support component sizing and positioning. The preoperative plan specified a Pinnacle acetabular cup (size 50) and a Summit femoral stem (size 6). Intraoperatively, the implanted cup and stem sizes fully matched the preoperative plan. Postoperative radiographs confirmed satisfactory component positioning and restoration of limb length (Figure 1).

#### 3.5.2 Post-septic HOA case

**FIGURE 2:**
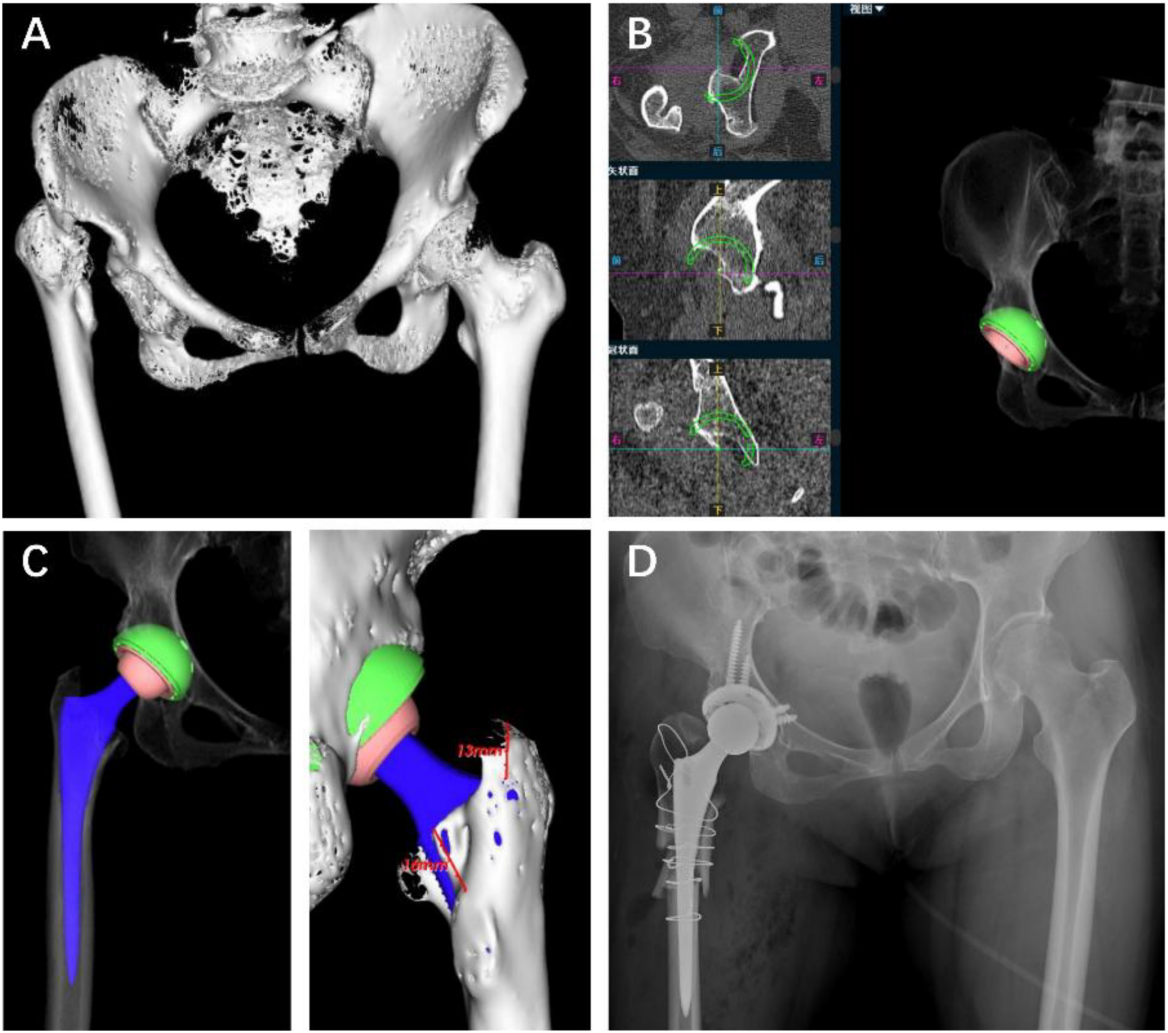
AI-assisted 3D preoperative planning in a Crowe IV post-septic HOA case. **(A)** 3D CT reconstruction. **(B)** Planned acetabular cup (Pinnacle, size 44). **(C)** Planned femoral stem (Summit, size 1), with the template exceeding the femoral bony envelope. **(D)** Postoperative radiograph showing the final position of the planned implants.

A 70-year-old woman underwent primary THA for post-septic HOA (Crowe type IV). CT-based, AI-assisted 3D preoperative planning proposed a Pinnacle acetabular cup (size 44) and a Summit femoral stem (size 1). Preoperative planning suggested limited femoral intramedullary accommodation, as the smallest stem template exceeded the available femoral bony envelope (Figure 2C), prompting preparation for adjunctive femoral reconstruction. A femoral osteotomy was performed, and the implanted component sizes fully matched the preoperative plan. Postoperative radiographs demonstrated appropriate implant position and restoration of limb length (Figure 2).

## 4 Discussion

Using a standardized CT-based, AI-assisted 3D planning pathway and a single cementless implant system, this study compared etiology-specific perioperative burden and recovery after primary THA for DDH-HOA versus post-septic HOA. Planning-to-implant size concordance, radiographic target attainment (Lewinnek/Callanan safe zones), and postoperative LLD were similarly high across etiologies. However, perioperative burden differed mainly in intraoperative blood loss, which remained higher in post-septic HOA, whereas operative time and the need for intraoperative femoral osteotomy were comparable. Clinically, DDH-HOA achieved better pain relief and functional recovery at 1 month, while the primary endpoint (HHS at 24 months) was comparable and PROMs largely converged by 24 months. These findings suggest that, despite standardizing key bony reconstruction targets within an AI-assisted pathway, etiology-related soft-tissue constraints and bone loss in post-septic sequelae may still translate into greater perioperative burden and slower early recovery (6, 16).

Prior literature in complex THA, particularly adult high dislocation, has reported a higher perioperative burden in post-septic sequelae than in DDH-related disease (6). In the present cohort, post-septic HOA showed higher blood loss despite the same CT-based AI-3D planning pathway and a single implant system, and this difference persisted despite comparable osteotomy rates between groups. This pattern is mechanistically plausible because AI-3D planning primarily optimizes osseous templating and component positioning, while exposure, reduction, and tension control can still be constrained by periarticular scarring, contracture, and segmental bone loss after infection. In practical terms, dense scar tissue and altered tissue planes may necessitate more extensive scar excision, soft-tissue release, and debridement to obtain adequate exposure and achieve a tension-balanced reduction, which can increase bleeding propensity. Matched comparative studies in Crowe IV reconstructions similarly reported greater blood loss and higher early complication rates in the post-septic setting (7, 23). Although prior septic arthritis has been associated with increased complications after THA, including PJI and subsequent reoperation in some series (4, 24), no deep infection or reoperation occurred within 24 months in the current study. This likely reflects stringent selection of clinically quiescent cases and a uniform perioperative protocol.

The slower early recovery observed in post-septic HOA is likely multifactorial. Residual bone loss and dense periarticular scarring with contracture may limit early range of motion and gait restoration, and potential abductor insufficiency may further compromise early function (25). The higher intraoperative blood loss in the post-septic cohort may also delay mobilization and rehabilitation. Comparative studies of high-dislocation reconstructions have reported greater perioperative burden and higher early complication risk in post-septic hips than in DDH-associated disease (6, 24). In addition, systematic reviews and case-control evidence indicate that a history of hip infection is associated with a less favorable early recovery profile after THA compared with non-infectious etiologies (4, 23). Consistent with these observations, between-group differences in pain and function attenuated by 24 months in the present cohort, supporting preoperative counseling that early recovery may be slower in post-septic HOA despite broadly comparable mid-term outcomes.

These results help delineate the role and limits of AI-assisted 3D planning in complex secondary HOA. Despite standardized bony targets and radiographic alignment across etiologies, post-septic HOA often requires additional soft-tissue releases and intraoperative adaptations to achieve adequate exposure and a tension-balanced reduction (26, 27). Accordingly, even with standardized bony targets, residual soft-tissue constraints after infection may continue to influence operative complexity and early outcomes, and may increase susceptibility to neurological injury, as reflected by the sciatic nerve injuries observed in the post-septic cohort.

From a practical perspective, perioperative counseling and preparation should remain etiology-specific even within a standardized AI-assisted pathway. For DDH-HOA, counseling should emphasize true acetabular reconstruction and, when indicated, subtrochanteric femoral shortening osteotomy, with attention to leg-length restoration and neural traction risk. For post-septic HOA, patients should be informed about a potentially more demanding reconstruction due to scarring, contracture, and bone loss, as well as a slower early recovery. Given the higher blood loss and the occurrence of sciatic nerve injury observed in the post-septic cohort, perioperative strategies should prioritize blood management, controlled reduction with careful tension assessment, and structured postoperative neurological monitoring (4, 24).

Several limitations should be acknowledged. First, the single-center retrospective design and modest sample size limit power for uncommon events and cannot exclude residual confounding. Second, the long inclusion period may introduce temporal bias, and we did not perform a formal early-versus-late comparison to quantify potential learning-curve effects. Third, follow-up was restricted to 24 months, precluding assessment of longer-term implant survivorship, late complications, and reoperation risk. Fourth, without a conventional two-dimensional templating comparator, the incremental benefit of CT-based AI-assisted planning over standard practice within each etiological subgroup could not be established. Fifth, potentially influential variables were not quantified in a standardized manner, including the extent of scarring/contracture, prior hip procedures, abductor status, and pathogen-related factors in the post-septic cohort. Radiation exposure and economic costs were also not evaluated. Finally, PROMs were compared at each time point without formal group-by-time interaction testing; therefore, the findings should be interpreted as associations rather than causal effects, consistent with STROBE reporting (28).

In summary, within a unified CT-based, AI-assisted 3D planning pathway and a single cementless implant system, etiology remained associated with perioperative burden and early recovery after primary THA for secondary HOA. Despite similarly high planning-to-implant concordance and radiographic target attainment, post-septic HOA was associated with greater blood loss and slower early improvement at 1 month, whereas PROMs largely converged by 24 months. Future multicentre prospective studies with longer follow-up are warranted to define long-term complications and implant survivorship and to incorporate quantifiable infection-related risk and soft-tissue status into etiology-sensitive risk stratification and perioperative care pathways for complex hip reconstruction (15, 26).

## 5 Conclusion

Within a unified CT-based AI-assisted 3D preoperative planning workflow and a single cementless implant system, component sizing concordance, cup orientation target attainment, and leg-length restoration were similarly high in DDH-HOA and post-septic HOA. By 24 months, PROMs were broadly comparable between groups. Post-septic sequelae were associated with greater intraoperative blood loss and less favorable pain and functional recovery at 1 month, while PROMs largely converged by 24 months. These findings support etiology-informed perioperative counseling and targeted blood-management and early rehabilitation strategies, and warrant validation in larger prospective cohorts with longer follow-up.

## Data Availability

The raw data supporting the conclusions of this article will be made available by the authors, without undue reservation.
